# Heterogeneous Nuclear Ribonucleoprotein E2 (hnRNP E2) Is a Component of TDP-43 Aggregates Specifically in the A and C Pathological Subtypes of Frontotemporal Lobar Degeneration

**DOI:** 10.3389/fnins.2019.00551

**Published:** 2019-06-04

**Authors:** Wejdan Kattuah, Boris Rogelj, Andrew King, Christopher E. Shaw, Tibor Hortobágyi, Claire Troakes

**Affiliations:** ^1^London Neurodegenerative Diseases Brain Bank, Department of Basic and Clinical Neuroscience, Institute of Psychiatry, Psychology and Neuroscience, King’s College London, London, United Kingdom; ^2^Department of Physiological Sciences, College of Medicine, Alfaisal University, Riyadh, Saudi Arabia; ^3^Department of Biotechnology, Jozef Stefan Institute, Ljubljana, Slovenia; ^4^Biomedical Research Institute BRIS, Ljubljana, Slovenia; ^5^Faculty of Chemistry and Chemical Technology, University of Ljubljana, Ljubljana, Slovenia; ^6^Department of Clinical Neuropathology, King’s College Hospital NHS Foundation Trust, London, United Kingdom; ^7^UK Dementia Research Institute, Department of Basic and Clinical Neuroscience, Maurice Wohl Clinical Neuroscience Institute, Institute of Psychiatry, Psychology and Neuroscience, King’s College London, London, United Kingdom; ^8^Department of Old Age Psychiatry, Institute of Psychiatry, Psychology and Neuroscience, King’s College London, London, United Kingdom; ^9^Department of Pathology, University of Szeged, Szeged, Hungary; ^10^MTA-DE Cerebrovascular and Neurodegenerative Research Group, Department of Neurology, University of Debrecen, Debrecen, Hungary

**Keywords:** FTLD, ALS, TDP-43, hnRNP, hnRNP E2

## Abstract

TAR DNA-binding protein 43 (TDP-43) is the major component of the ubiquitin-positive protein aggregates seen in the majority of frontotemporal lobar degeneration and amyotrophic lateral sclerosis cases. TDP-43 belongs to the heterogeneous nuclear ribonucleoprotein (hnRNP) family that is involved in the regulation of RNA transcription, splicing, transport and translation. There are a great many hnRNPs, which often have overlapping functions and act cooperatively in RNA processing. Here we demonstrate that another hnRNP family member, hnRNP E2, shows a striking accumulation within dystrophic neurites and cytoplasmic inclusions in the frontal cortex and hippocampus of a subset of FTLD-TDP cases belonging to pathological subtypes A and C, where hnRNP E2 was found to co-localize with 87% of TDP-43 immunopositive inclusions. hnRNP E2-positive inclusions were not seen in FTLD-TDP cases with the *C9orf72* expansion or in any other neurodegenerative disorders examined. This interaction with TDP-43 in specific FTLD subtypes suggests different underlying neurodegenerative pathways.

## Introduction

Frontotemporal lobar degeneration (FTLD) refers to a group of neurodegenerative disorders predominantly affecting the frontal and temporal lobes. The group is clinically, pathologically and genetically heterogeneous. Typically, patients initially present with behavioral dysfunction and changes in personal and social conduct (behavioral variant) or with disorders of speech and language (semantic dementia and progressive non-fluent aphasia). FTLD can occur alone or, as seen in approximately 15% of cases, accompanied by amyotrophic lateral sclerosis (ALS) with progressive upper and lower motor neuron degeneration.

Approximately 30–50% of FTLD cases are familial with an autosomal dominant pattern of inheritance. Reported mutations include *MAPT*, *GRN*, *VCP* and the GGGGCC repeat expansion in the *C9orf72* gene (Watts et al., 2004; Baker et al., 2006; DeJesus-Hernandez et al., 2011).

Pathologically, FTLD cases exhibit intraneuronal (and in some cases glial) inclusions formed of abnormally aggregated proteins. Cases can be classified according to the type of hallmark protein accumulated in the neuronal inclusions, with most cases showing inclusions positive for either tau (FTLD-tau) or TDP-43 (FTLD-TDP) and, less frequently, FUS (FTLD-FUS).

Since 2011, a harmonized classification has been used in FTLD-TDP cases based on the morphology and neuroanatomical distribution of inclusions (Mackenzie et al., 2011). Briefly, FTLD-TDP type A is characterized by crescentic or oval neuronal cytoplasmic inclusions (NCI) and numerous short dystrophic neurites (DN), primarily in layer 2 of the neocortex. Lentiform neuronal intranuclear inclusions (NII) can sometimes be seen, however, they are not a consistent feature of this subtype. Type B is characterized by a moderate number of NCI in all cortical layers and very few DN. Type C have numerous elongated DN in the upper cortical layers but very few NCI. Type D FTLD-TDP refers to the pathology associated with *VCP* mutations and is characterized by frequent lentiform NII and numerous short DN. The pathological subtypes of FTLD-TDP cases have some correlation to the clinical phenotypes, subtype A being associated with behavioral variant of frontotemporal dementia (bvFTD) or progressive non-fluent aphasia (PFNA), subtype B with bvFTD and often combined with ALS and subtype C with semantic dementia. Subtype D is often associated with Paget’s disease of bone and hereditary inclusion body myopathy

Normally TDP-43 is predominantly localized to the nucleus, however, it is continuously shuttling between the nucleus and the cytoplasm (Ayala et al., 2008; Prpar Mihevc et al., 2017). It is capable of binding to nucleic acids, with involvement in RNA splicing, stability, transcription and translation (Da Cruz and Cleveland, 2011; Tollervey et al., 2011; Buratti and Baralle, 2012). TDP-43 is a member of the heterogeneous ribonucleoprotein (hnRNP) family and interacts with a number of other members, mainly through its C-terminal tail (Romano et al., 2014). Mutations in TDP-43 have been associated with ALS, but not FTLD (Sreedharan et al., 2008).

Other hnRNPs have previously been reported to be associated with pathology in FTLD-TDP. hnRNP A3 was identified as a component of some of the p62-positive and TDP-43-negative hippocampal inclusions seen in a subset of FTLD/ALS cases with the *C9orf72* expansion; it was also shown to be a component of “RNA foci” and it has been suggested that it binds to the GGGGCC repeats in *C9orf72* transcripts (Mori et al., 2013); however, its pathogenic role has not yet been determined. In addition, the recent implication of hnRNP A2/B1 and hnRNP A1 in ALS and multisystem proteinopathy supports the hypothesis of a physical and functional interaction between TDP-43 and other hnRNPs (Calini et al., 2013; Kim et al., 2013; Romano et al., 2014). The relative expression of a specific protein within the TDP-43 interaction network may have a significant impact on the function of TDP-43, either through direct interaction or independently by acting on the same cellular targets (Hanson et al., 2010; Buratti and Baralle, 2012; Mohagheghi et al., 2016).

hnRNP E2 is an RNA-binding protein that belongs to the hnRNP K family of proteins. They are characterized by triple K Homology (KH) domains that can interact independently with target RNA sequences, which allows this protein family to potentially form highly complex-specific RNA interactions. hnRNP E2 has been reported to incorporate into stress granules alongside TDP-43 (Fujimura et al., 2008; Liu-Yesucevitz et al., 2010) and in a recent pathological study hnRNP E2 was shown to colocalise with TDP-43 inclusions in FTLD cases which presented with the semantic dementia clinical phenotype (Davidson et al., 2017).

Here we examined the expression of hnRNP E2 in a large cohort of cases, including a FTLD-TDP cohort comprising cases from all 4 pathological subtypes, cases with and without the *C9orf72* expansion, controls, and a number of other neurodegenerative diseases.

## Materials and Methods

### Case Selection and Tissue Preparation

Post-mortem brain tissue samples in 10% formalin-fixed paraffin embedded blocks were obtained from the MRC London Neurodegenerative Diseases Brain Bank at the Institute of Psychiatry, Psychology and Neuroscience, King’s College London. Consent for autopsy, neuropathological assessment and research was obtained for all cases and the study was carried out under the ethical approval of the tissue bank. Block taking and neuropathological assessment was performed according to standard criteria.

Tissue samples were selected from a total of 108 cases. 30 cases of FTLD-TDP without the *C9orf72* expansion (FTLD-TDP) were examined (comprising 15 classified as pathological subtype A, four subtype B, nine subtype C, one subtype D case and one which could not be definitively classified due to atypical inclusions). Additionally, 24 FTLD-TDP cases with *C9orf72* expansion were investigated (FTLD-TDP-*C9orf72*) (of which three were identified as subtype A, 16 as subtype B, and five which could not be definitively subtyped).

To investigate the specificity of hnRNP E2 to FTLD-TDP, seven FTLD with tau aggregates (FTLD-Tau), 14 sporadic ALS (sALS), three ALS with *SOD1* mutations (ALS-*SOD*), three ALS with *FUS* mutations (ALS-*FUS*) and one ALS with a *TARDBP* mutation were examined.

To determine the specificity of hnRNP E2 to FTLD, seven Alzheimer’s disease, two Dementia with Lewy bodies (DLB), one argyrophilic grain disease (AGD), one spinocerebellar ataxia (SCA) and one Huntington’s disease case were examined. Fourteen healthy controls without a history of neurological problems or psychiatric disorders and without any significant pathology (matched for gender, age and post-mortem delay) were also investigated (see Table 1 for details).

**Table 1:** List of cases used in the study, showing demographic details and hnRNP E2 immunohistochemistry positivity.

MRC ID	Diagnosis	TDP-Subtype	Sex	Age at death	PMD	Fixation time	hnRNP E2 positivity		
							Frontal	H/C	SC
							
BBN_1085	FTLD-TDP	A	M	87	41	12	N	N	N/A
BBN_15298	FTLD-TDP (?ALS)	A	M	69	42	5	**Y**	**Y**	**Y**
BBN_10599	FTLD-TDP	A	F	79	56	6	**Y**	N	N/A
BBN_4568	FTLD-TDP	A	M	59	36	4	**Y**	**Y**	**Y**
BBN_15299	FTLD-TDP	A	M	88	31	8	**Y**	N	N/A
BBN_15306	FTLD-TDP	A	M	71	14	8	N	N	N
BBN_10245	FTLD-TDP	A	M	87	31	10	**Y**	N	N
BBN_9863	FTLD-TDP	A	M	73	25	8	N	N	N/A
BBN_15281	FTLD-TDP	A	F	67	15	7	N	N	N/A
BBN_19697	FTLD-TDP	A	F	78	72	8	N	N	N/A
BBN_15302	FTLD-TDP	A	M	81	11	24	N	N	N/A
BBN_16282	FTLD-TDP	A	M	78	>100	4	N	N	N/A
BBN_15292	FTLD-TDP	A	F	56	35	10	**Y**	N	N/A
BBN_15287	FTLD-TDP	A	F	74	70	8	N	N	N/A
BBN_15283	FTLD-TDP	A	M	70	57	22	N	N	N/A
BBN_9950	FTLD-TDP	B	M	72	38	12	N	N	N/A
BBN_4590	FTLD-TDP	B	F	73	54	8	N	N	N
BBN_15289	FTLD-TDP	B	M	68	46	4	N	N	N
BBN_11067	FTLD-TDP	B	M	81	31	8	N	N	N/A
BBN_15303	FTLD-TDP	C	M	69	6	12	**Y**	**Y**	N
BBN_15286	FTLD-TDP	C	M	68	120	7	**Y**	**Y**	N
BBN_15200	FTLD-TDP	C	M	69	16	11	**Y**	**Y**	N
BBN_15294	FTLD-TDP	C	F	85	24	11	**Y**	**Y**	N/A
BBN_15304	FTLD-TDP	C	M	80	45	20	**Y**	**Y**	N
BBN_15295	FTLD-TDP	C	M	82	14	16	**Y**	**Y**	**Y**
BBN_15297	FTLD-TDP	C	M	78	24	12	**Y**	**Y**	N/A
BBN_15288	FTLD-TDP	C	M	80	7	12	**Y**	**Y**	N/A
BBN_15290	FTLD-TDP	C	M	66	54	8	**Y**	**Y**	N
N/A	FTLD-TDP	D	M	61	3	14	N	N	N
BBN_4249	FTLD-TDP	NC	M	86	45	6	**Y**	N	N
BBN_15300	FTLD-ALS *C9orf72*	A	M	79	35	5	N	N	N/A
BBN_15291	FTLD-ALS *C9orf72*	A	M	56	19	11	N	N	N/A
BBN_16438	FTLD-ALS *C9orf72*	A	F	58	12	61	N	N	N/A
BBN_15279	FTLD-*C9orf72*	B	F	57	16	18	N	N	N
BBN_6230	FTLD-ALS *C9orf72*	B	M	71	44	17	**Y**	N	**Y**
BBN_15296	FTLD-*C9orf72*	B	F	70	16	12	N	N	N
BBN_16615	FTLD-ALS *C9orf72*	B	F	64	44	4	N	N	N/A
BBN_15713	FTLD-ALS *C9orf72*	B	M	57	23	35	N	N	N/A
BBN_6252	FTLD-ALS *C9orf72*	B	F	43	69	14	N	N	N/A
BBN_6254	FTLD-ALS *C9orf72*	B	M	53	82	10	N	N	N/A
BBN_16304	FTLD-ALS *C9orf72*	B	M	59	46	10	N	N	N/A
BBN_16651	FTLD-ALS *C9orf72*	B	M	51	64	7	N	N	N/A
BBN_16458	FTLD-ALS *C9orf72*	B	M	70	40	8	N	N	N/A
BBN_6227	FTLD-ALS *C9orf72*	B	M	55	76	31	N	N	N/A
BBN_6198	FTLD-ALS *C9orf72*	B	M	58	11	15	N	N	N/A
BBN_10306	FTLD-ALS *C9orf72*	B	M	64	68	11	N	N	N/A
BBN_16969	FTLD-ALS *C9orf72*	B	F	57	12	12	N	N	N/A
BBN_4253	FTLD-ALS *C9orf72*	B	F	59	21	10	N	N	N/A
BBN_6251	FTLD-ALS *C9orf72*	B	M	62	74	14	N	N	N/A
BBN_16380	FTLD-ALS *C9orf72*	NC	F	59	35	17	N	N	N/A
BBN_16223	FTLD-ALS *C9orf72*	NC	M	55	19	17	N	N	N/A
BBN_6242	FTLD-ALS *C9orf72*	NC	F	39	70	18	N	N	N/A
BBN_6232	FTLD-ALS *C9orf72*	NC	M	70	38	12	N	N	N/A
BBN_15641	FTLD-ALS *C9orf72*	NC	F	70	60	37	N	N	N/A

BBN_15268	FTLD-Tau		F	58	31	13	N	N	N/A
BBN_15269	FTLD-Tau		M	67	35	9	N	N	N/A
BBN_15284	FTLD-Tau		F	62	31	9	N	N	N/A
BBN_10282	FTLD-Tau		M	72	6	19	N	N	N/A
BBN_10281	FTLD-Tau		M	61	23	21	N	N	N/A
BBN_15776	FTLD-Tau		M	66	17	3	N	N	N/A
BBN_15285	FTLD-Tau		M	67	17	7	N	N	N/A
BBN_6244	sALS		M	55	33	22	N	N	N/A
BBN_6187	sALS		M	70	73	75	N	N	N/A
BBN_10272	sALS		M	87	70	22	N	N	N/A
BBN_6248	sALS		M	66	38	8	N	N	N/A
BBN_15715	sALS		M	74	34	40	N	N	N/A
BBN_6219	sALS		M	49	33	33	N	N	N/A
BBN_6267	sALS		M	68	5	18	N	N	N/A
BBN_6257	sALS		F	69	64	14	N	N	N/A
BBN_6243	sALS		M	67	70	11	N	N	N/A
BBN_6217	sALS		F	56	39	56	N	N	N/A
BBN_6268	sALS		M	78	2	18	N	N	N/A
BBN_6280	sALS		M	75	38	9	N	N	N/A
BBN_10285	sALS		M	42	41	23	N	N	N/A
BBN_16384	sALS		F	57	15	23	N	N	N/A
BBN_16392	ALS-*SOD*		F	61	14	21	N	N	N/A
BBN_10276	ALS-*SOD*		M	47	14	18	N	N	N/A
BBN_16553	ALS-*SOD*		F	46	5	14	N	N	N/A
BBN_6245	ALS-*FUS*		F	35	13	5	N	N	N/A
BBN_6189	ALS-*FUS*		F	35	24	39	N	N	N/A
BBN_10244	ALS-*FUS*		F	23	37	12	N	N	N/A
BBN_19995	ALS-*TARDBP*		M	57	48	9	N	N	N/A
BBN_9933	Alzheimer’s Disease		F	98	25	9	N	N	N/A
BBN_9934	Alzheimer’s Disease		M	70	60	19	N	N	N/A
BBN_4182	Alzheimer’s Disease		F	81	23	18	N	N	N/A
BBN_4183	Alzheimer’s Disease		F	79	40	8	N	N	N/A
BBN_9801	Alzheimer’s Disease		F	90	23	20	N	N	N/A
BBN_9927	Alzheimer’s Disease		F	90	35	18	N	N	N/A
BBN_9930	Alzheimer’s Disease		M	101	48	12	N	N	N/A
BBN_16337	DLB		M	85	30	18	N	N	N/A
BBN_10290	DLB		M	78	41	25	N	N	N/A
BBN_2924	Agyrophilic Grain Disease		M	82	20	8	N	N	N/A
BBN_15766	SCA		F	74	33	53	N	N	N/A
BBN_11070	Huntington’s disease		M	65	36	8	N	N	N/A
BBN_15777	Control		F	87	22	150	N	N	N/A
BBN_15753	Control		M	64	71	53	N	N	N/A
BBN_16291	Control		M	81	18	17	N	N	N/A
BBN_16242	Control		F	90	50	19	N	N	N/A
BBN_15621	Control		M	61	53	14	N	N	N/A
BBN_16429	Control		M	68	53	4	N	N	N/A
BBN_16280	Control		M	78	24	8	N	N	N/A
BBN_16277	Control		M	54	30	8	N	N	N/A
BBN_15791	Control		M	95	44	20	N	N	N/A
BBN_15790	Control		M	40	40	25	N	N	N/A
BBN_16256	Control		M	62	80	18	N	N	N/A
BBN_16251	Control		M	66	52	7	N	N	N/A
BBN_22991	Control		F	73	27	6	N	N	N/A
BBN_16525	Control		F	77	29	14	N	N	N/A

*PMD (post-mortem delay), H/C (hippocampus), SC (spinal cord) ?ALS (sparse pathology seen in cord but no clinical symptoms reported), sALS (sporadic ALS), DLB (Dementia with Lewy bodies), SCA (Spinocerebellar ataxia), NC (non-classifiable), N/A (not available). Y (hnRNP-E2 positive aggregates).*

In each case frontal and temporal lobe (containing the hippocampus) were examined. In some cases (where available) the spinal cord was also studied.

### Immunohistochemistry

Seven micrometer thick, formalin fixed, paraffin embedded sections were cut from the middle frontal gyrus, hippocampus and, in some cases, the spinal cord. Immunohistochemistry was conducted as per previously published protocols (Maekawa et al., 2009). In brief, sections were deparaffinised in xylene and endogenous peroxidase was blocked by immersion in 2.5% H_2_O_2_ in methanol. Antigen retrieval was enhanced using an extended microwave citrate buffer treatment. After blocking in normal swine serum (DAKO, Cambridgeshire, United Kingdom) 1:10 for 20 min, hnRNP E2 antibody (hnRNP E2-23G, sc-101136, Santa Cruz) was applied at 1:500 overnight at 4°C. Following washes, the sections were incubated with biotinylated secondary antibody (DAKO), followed by avidin:biotinylated enzyme complex (Vectastain Elite ABC kit, Vector Laboratories, Peterborough, United Kingdom). The sections were then incubated for 10–15 min with 0.5 mg/mL 3,3′-diaminobenzidine chromogen (Sigma-Aldrich Company Ltd., Dorset, United Kingdom) in Tris-buffered saline (pH 7.6) containing 0.05% H_2_O_2_. The sections were counterstained with Harris’ haematoxylin and immunostaining analyzed using a Leica microscope.

### Double Immunofluorescence

To investigate co-localisation of hnRNP E2 with ubiquitin and TDP-43, double immunofluorescence was carried out on a subset of the FTLD-TDP cases. Seven micrometer sections cut from formalin-fixed paraffin-embedded blocks were deparaffinised in xylene and dehydrated in 99% industrial methylated spirit. Sections were pre-treated with microwave heating in citrate buffer and normal serum blocking was performed using normal goat serum (1:10 for 45 min). Sections were incubated overnight at 4°C with hnRNP E2 (hnRNP E2-23G: sc-101136, Santa Cruz) at 1:200 with either TDP-43 (Proteintech, 10782-2-AP) at 1:250 or anti-ubiquitin (Dako, Z045801) at 1:100.

After washes, sections were incubated with AlexaFluor secondary antibodies (goat anti-mouse 488 and goat anti-rabbit 568, Invitrogen, Paisley, United Kingdom). Sections were treated with Sudan Black for 10 min to quench autofluorescence. Following numerous washes in phosphate buffered saline, sections were mounted with Vectashield hard set media containing DAPI. Sections were visualized using a fluorescent microscope (Zeiss Axiovert S 100, Gottingen, Germany) and images captured using ImagePro Express (v6) (Meyer Instruments, Houston, TX, United States). Sections were also examined using confocal microscopy (Leica confocal SP system) and captured images analyzed using ImageJ 1.47v software.

### Statistical Analysis of hnRNP-E2 Pathology and Demographic Data

A Chi-square test of independence was performed to examine the relationship between gender, age at death, post-mortem delay and fixation time with presence of hnRNP E2 pathology.

## Results

**FIGURE 1:**
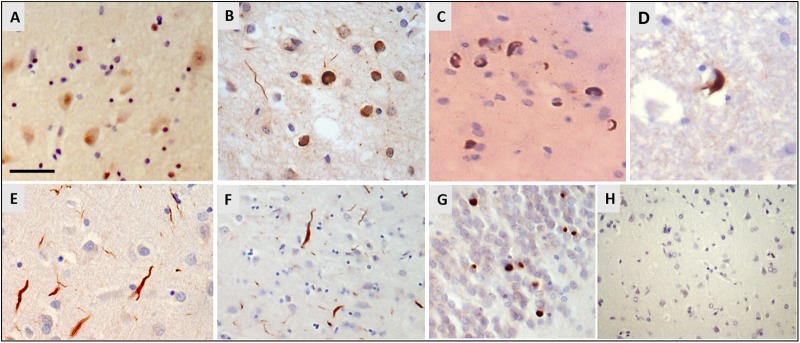
Immunohistochemistry for hnRNP E2. **(A)** Weak cytoplasmic staining and stronger nuclear staining is seen in the frontal cortex of a control case (case ID BBN_16256). **(B–D)** Sections from a FTLD-TDP subtype A case showed numerous perinuclear cytoplasmic neuronal inclusions and frequent dystrophic neurites, mainly in layer 2 of the frontal cortex **(B)**. Similar cytoplasmic inclusions were detected in hippocampal neurons **(C)** and sparsely in the anterior horn of the spinal cord **(D)** (case ID BBN_15298). **(E–G)** In FTLD-TDP subtype C there were frequent long dystrophic neurites in the superficial layers of the frontal cortex **(E)** and in the hippocampus **(F)**. Within the granular cells of the dentate fascia round intracellular inclusions were seen **(G)** (case ID BBN_15303). **(H)** No inclusions were seen in the frontal cortex of the subtype D case **(H)**. Scale bar represents 20 μm in panels **(A,B,E)**; 80 μm in panels **(C,D,G)**; 100 μm in panel **(F,H)**.

Immunohistochemistry for hnRNP E2 showed striking inclusions in 17 FTLD-TDP cases, specifically those with subtype A and C pathology (see Table 1). The inclusions showed a similar pattern to that of the TDP-43 pathology. In subtype A FTLD-TDP cases six out of 15 cases showed hnRNP E2 inclusions in the frontal cortex with two of these also showing inclusions in the hippocampus and in the spinal cord. Within the frontal cortex hnRNP E2 inclusions were predominant in the superficial layers, mainly in layer 2 of the neocortex, with numerous perinuclear cytoplasmic neuronal inclusions and frequent dystrophic neurites (Figure 1B). Similar inclusions were detected within the hippocampus granular cells (Figure 1C). In the spinal cord, sparse inclusions were detected in the anterior horn of the thoracic spinal cord in the two cases (Figure 1D). All nine subtype C cases showed hnRNP E2 inclusions in the frontal cortex; long dystrophic neurites were predominantly detected in the superficial layers of the frontal cortex (Figure 1E) and in the hippocampus (Figure 1F). In all nine cases round intracellular inclusions were also detected in the granular cells of dentate fascia (Figure 1G) and in one case inclusions were seen in the spinal cord. hnRNP E2-positive inclusions were seen in both the frontal cortex and the spinal cord of an additional FTLD-TDP case that, due to atypical p62 positive, TDP negative intranuclear inclusions, could not be definitively classified into a sub-group. No hnRNP E2 positive inclusions were seen in either the frontal cortex or hippocampus of the FTLD-TDP subtype D case (Figure 1H). Inclusions were seen in just one of the FTLD-TDP C9orf72 expansion positive cases (classified as a subtype B). Within control cases, the hnRNP E2 immunohistochemistry showed weak diffuse staining in the cytoplasm and stronger nuclear staining (Figure 1A). No hnRNP E2 aggregates were seen in the brain or spinal cord in any disease other than FTLD-TDP, with staining appearing similar to the control cases.

### Co-localisation of TDP-43 and hnRNP E2

**FIGURE 2:**
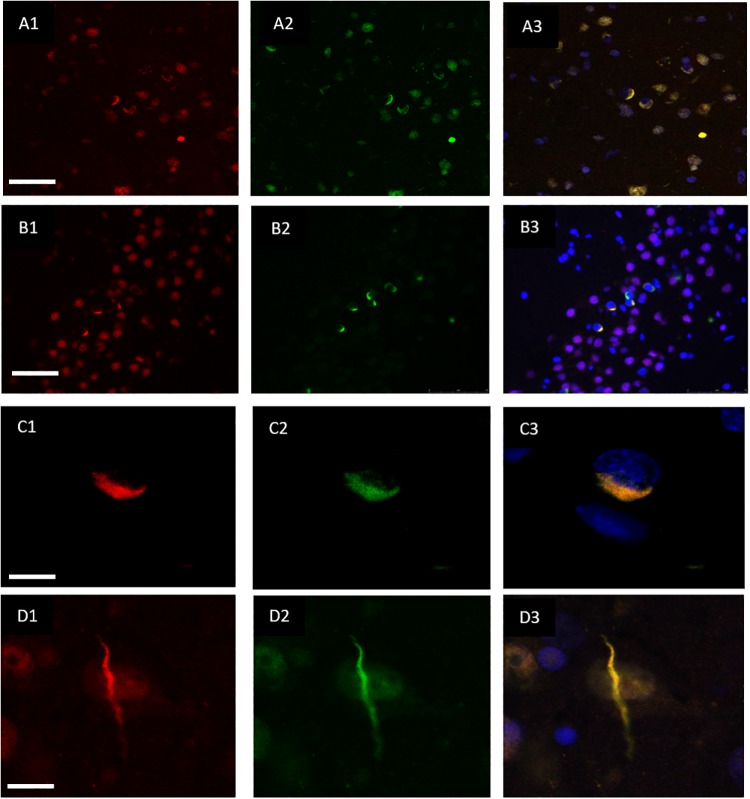
Co-localisation of TDP-43 and hnRNP E2. Double-labeling immunofluorescence shows inclusions positive for TDP-43 (red) **(A1)** and hnRNP E2 (green) **(A2)** in the frontal cortex of a subtype A case, the merged image **(A3)** shows numerous areas of co-localisation (case ID BBN_15298). Co-localisation is also seen in the hippocampus, panels **(B1–B3)** show a subtype A case (case ID 482 BBN_4568). Higher magnification images demonstrate the complete co-localisation of the TDP-43 and hnRNP E2 in a perinuclear inclusion from the subtype A case **(C1–C3)** and along a dystrophic neurite in a subtype C case **(D1–D3)** (case ID BBN_15303). Scale bar represents 100 μm in panels **(A1–A3,B1–B3)**, 25 μm in panels **(C1–C3,D1–D3)**.

Double immunofluorescence revealed a strong relationship between TDP-43 and hnRNP E2, in both the frontal cortex (Figure 2A1–A3) and hippocampus (Figure 2B1–B3) with 87% of the TDP-43-positive inclusions being found to co-localize with hnRNP E2 (a total of 583 TDP-43 positive inclusions and 507 hnRNP E2 positive inclusions were counted in three FTLD-TDP type A cases and four FTLD-TDP type C cases). Higher magnification images of individual cells were obtained using a confocal microscope, which showed the complete co-localisation of both TDP-43 and hnRNP E2 in the perinuclear inclusions and dystrophic neurites, respectively (Figure 2C1–C3). Images were also obtained from a type C FTLD-TDP case where mainly TDP-43 dystrophic neurites are detected in the frontal cortex, again, complete co-localisation of hnRNP E2 with TDP-43 was detected (Figure 2D1–D3). Interestingly the *C9orf72* positive case showed little co-localisation between hnRNP E2 and TDP-43 (image not shown).

### Co-localisation of Ubiquitin and hnRNP-E2

**FIGURE 3:**
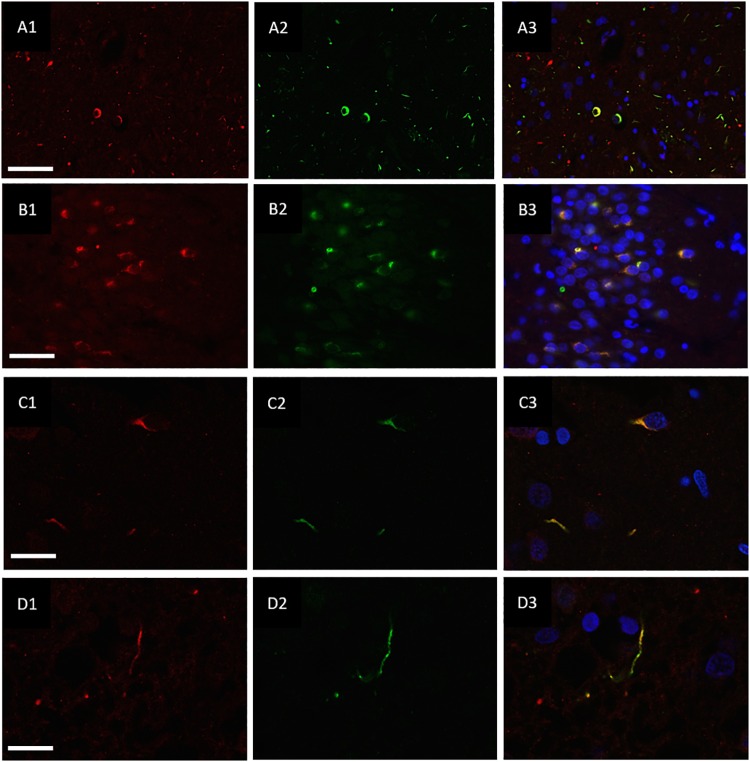
Co-localisation of ubiquitin and hnRNP E2. Double-labeling immunofluorescence of Ubiquitin (red) and hnRNP E2 (green) shows high levels of co-localisation within the frontal cortex **(A1–A3)** and hippocampus **(B1–B3)** of a subtype A case (case ID BBN_4568). Strong co-localisation can be seen within the inclusions in a subtype A case **(C1–C3)** (case ID BBN_15298). In a subtype C case, some inclusions show only ubiquitin positivity **(D1)** and some only hnRNP-E2 positivity **(D2)**. Within inclusions showing both proteins, the co-localisation is not absolute **(D3)** (case ID BBN_15303). Scale bar represents 80 μm in panel **(A1–A3)**, 50 μm in panel **(B1–B3)**, 20 μm in panels **(C1–C3,D1–D3)**.

hnRNP E2 was also present in 70% of the ubiquitin-positive inclusions in the frontal cortex (Figure 3A1–A3) and hippocampus (Figure 3B1–B3) of the same cases (with a total of 810 ubiquitin positive inclusions and 571 hnRNP-E2 positive inclusions being counted from three FTLD-TDP type A cases and four FTLD-TDP type C cases). High resolution images of the hnRNP E2/ubiquitin inclusions appear to show hnRNP E2 is partially ubiquitinated (Figure 3C1–C3,D1–D3).

### Effects of Gender, Age, PMD and Fixation Time

hnRNP E2 pathology was found to be independent of both gender and age. There was also no significant effect of PMD or fixation time on the presence of hnRNP E2 pathology in the FTLD-TDP cases (Table 2).

**Table 2:** No significant effect of gender, age, post-mortem delay or fixation time was seen on hnRNP E2 positivity in FTLD-TDP cases using a Pearson’s Chi-squared test (for gender) or Welch Two Sample *t*-test (for age, post-mortem delay and fixation time) df = degrees of freedom.

Variable	Test	Df	*p*-value
Gender	X-squared = 0.4026	1	0.5257
Age	*t* = −0.3908	26.293	0.6991
Post-mortem delay	*t* = 0.5136	26.736	0.6118
Fixation time	*t* = 0.3276	22.793	0.7462

## Discussion

Since its identification as the major component of the protein aggregates in the majority of ALS and FTLD cases, TDP-43 has been subject to much investigation. However, its mechanistic role in neuronal degeneration has not yet been fully characterized. Identifying proteins that interact with TDP-43 would represent a major step forward in understanding the pathological mechanism and pathways.

In this study we report that immunohistochemistry for hnRNP E2 revealed prominent perinuclear cytoplasmic inclusions and dystrophic neurites in FTLD-TDP patient tissue – specifically in those classified as pathological subtypes A (6/15) and C (9/9). Positive inclusions were also seen in an additional FTLD-TDP case which was unable to be exactly classified, as well as in just a single *C9orf72* expansion positive case [classed as subtype B – although it should be noted that the classification of *C9orf72* cases can sometimes be difficult (Mackenzie and Neumann, 2017)]. Inclusions were not seen in subtype B or D FTLD-TDP or in the majority of *C9orf72* expansion-positive cases, despite the presence of TDP-43 inclusions. This adds to the increasing evidence for differences in the underlying mechanisms driving TDP-43 aggregation in different subtypes of FTLD-TDP. It has been proposed that TDP-43 may not be the driving force behind the neurodegeneration seen in *C9orf72* expansion cases (Leko et al., 2019) although this remains controversial (Mackenzie et al., 2015). No inclusions were seen in control cases or in any other neurodegenerative condition investigated.

Further insight into the functions of hnRNP E2 within the nervous system could provide an explanation for the co-localisation of hnRNP E2 with TDP-43 in the inclusions. Recent evidence suggests one explanation could be the role of hnRNP E2 in the regulation of apoptosis. Evidence of neuronal apoptosis has been reported in ALS and FTLD-TDP cases, with elevated levels of activated caspase-3 seen in the spinal cord and brain (Martin, 1999; Su et al., 2000) and caspase-3 being identified as the protease responsible for TDP-43 fragmentation (Zhang et al., 2007). Interestingly, caspase-3 downregulates TDP-43 in glioma cells (Nan et al., 2014). Previous studies have linked hnRNP E2 to caspase-3 activation; hnRNP E2 is upregulated in human glioma tissue, while hnRNP E2 knockdown inhibited glioma growth through the induction of caspase-3-mediated apoptosis and the inhibition of cell-cycle progression (Han et al., 2013). Additionally, it has been reported that the overexpression of hnRNP E2 induces apoptosis in human oral cancer cells (Roychoudhury et al., 2007). Following spinal cord injury, both hnRNP E2 and caspase-3 were upregulated in neurons (Mao et al., 2016) and knockdown of hnRNP E2 decreased the expression of caspase-3 in primary neuronal cultures. However, the levels of cyclin D1 did not change after hnRNP E2 knockdown, which suggested that hnRNP E2-induced neuronal apoptosis is independent of cell cycle activation (Mao et al., 2016). The detailed mechanism of how hnRNP E2 may modulate caspase-3 activity and apoptosis has not yet been clarified but it is possible that this mechanism may mediate TDP-43 aggregation in some FTLD-TDP cases.

A further possible explanation for the co-localisation of hnRNP E2 and TDP-43 in the FTLD-TDP brain is their role in the stress response. It has been shown that TDP-43 and hnRNP E2 are both recruited into stress granules under stress conditions (Fujimura et al., 2008; Monahan et al., 2016). Following oxidative stress, endogenous TDP-43 and hnRNP E2 colocalised within stress granules as well as with the stress granule marker PABP1, an effect that was greatly enhanced by the sequential addition of an osmotic challenge. hnRNP E2 has been identified as a facilitator of internal ribosome entry site-mediated translation and to have an important role in remodeling mRNAs in the stress granules and in transferring specific mRNAs from stress granules to P-bodies for degradation (Fujimura et al., 2008, 2009).

Another possible link could be established through microRNAs (miRNA). Nuclear and cytoplasmic TDP-43 were identified as modulators of miRNA maturation and, by facilitating miRNA production, to be essential for neuronal outgrowth (Kawahara and Mieda-Sato, 2012). Interestingly, hnRNP E2 expression was found to be regulated by miRNA-214; however, the regulatory pathway still needs to be determined (Tang et al., 2015).

Both TDP-43 and hnRNP E2 are present alongside ubiquitin in FTLD-TDP cases, thus, it is possible that ubiquitin could act as the link between TDP-43 and hnRNP E2. Scotter et al. (2014) suggested that an impaired ubiquitin proteasome system (UPS) contributes to elevated TDP-43 levels. The cascade of ubiquitin-mediated protein degradation involves the stepwise action of three enzymes, namely the ubiquitin-activating enzyme (E1), the ubiquitin-conjugating enzyme (E2) and the ubiquitin ligase enzyme (E3), which provide substrate specificity. E3 ubiquitin ligase recruits the ubiquitin-loaded conjugating enzyme E2, recognizes a protein substrate and either directly catalyzes or assists with the transfer of ubiquitin from E2 to the protein substrate (Hershko and Ciechanover, 1998). hnRNP E2 was identified as an adapter between the ubiquitin ligase E3 and the mitochondrial antiviral-signaling protein (MAVS) in cellular studies (You et al., 2009). The overexpression of hnRNP E2 led to the degradation of MAVS and abolished the cellular response to viral infection, while the knockdown of hnRNP E2 had the opposite effect. hnRNP E2 is not a ligase enzyme; however, it performs a ligase-enzyme-adapting activity, which recruits the conjugating enzyme to its substrate (You et al., 2009). Moreover, a ubiquitin ligase complex has been recently identified that is involved in TDP-43 degradation (Uchida et al., 2016). The ligase complex consists of the von Hippel-Lindau protein (VHL) and the cullin-2 (CUL2) RING, which belongs to the hydrophobic family of proteins that provides a temporary complex for ubiquitin ligase E3. VHL preferential binds to the RRM2 of misfolded TDP-43. Interestingly, when VHL is overloaded in the cytoplasm, it tends to stabilize and aggregate with TDP-43. VHL was detected in TDP-43 inclusions in spinal cord motor neurons of ALS patients, suggesting that the imbalance between VHL and CUL2 is the key to TDP-43 aggregation and highlighted the CUL2 E3 ligase as a potential therapeutic target for TDP-43 proteinopathies (Uchida et al., 2016). Determining whether hnRNP E2 could be a component or an adaptor of this ligase complex requires further investigation.

Our work corroborates the findings of a recent study by Davidson et al. (2017), where hnRNP E2 inclusions were reported in a subset of FTLD-TDP subtype C only. The authors suggested that long-term storage of tissue in formalin might have been the cause of variations in hnRNP E2 staining among the FTLD-TDP type C group. Our study showed inclusions in all type C cases examined (9/9) as well as a proportion of type A (6/16). Statistical analysis did not find any significant effect of fixation time on presence of hnRNP E2 staining, nor was there any relationship between gender, age or post-mortem delay in the FTLD-TDP cases.

Our findings highlight the importance of characterizing the protein composition of aggregates in order to understand the underlying pathological mechanisms and suggest that the co-localisation of hnRNP E2 with TDP-43 in inclusions may have implications in the pathogenesis of a subset of FTLD-TDP cases - which could even be considered as a subtype in future classifications of FTLD-TDP-43 proteinopathies.

## Ethics Statement

This study was carried out in accordance with the Tissue Bank ethical approval for the London Neurodegenerative Diseases Brain Bank (18/WA/0206, Wales REC 3) with written informed consent from all subjects. All subjects gave written informed consent in accordance with the Declaration of Helsinki. The protocol was approved by the Wales REC 3.

## Author Contributions

WK and CT conducted the immunohistochemical and immunofluorescent staining. CT, TH, and CS designed the project. AK and TH conducted neuropathological assessment and subtyping of FTLD-TDP-43 cases. WK and CT wrote the manuscript. All authors reviewed and approved the final manuscript.

## Conflict of Interest Statement

The authors declare that the research was conducted in the absence of any commercial or financial relationships that could be construed as a potential conflict of interest.
